# Thermography as a non-ionizing quantitative tool for diagnosing periapical inflammatory lesions

**DOI:** 10.1186/s12903-021-01618-9

**Published:** 2021-05-13

**Authors:** M. Atef Aboushady, Wael Talaat, Zaid Hamdoon, Tarek M.Elshazly, Nivin Ragy, Christoph Bourauel, Sameh Talaat

**Affiliations:** 1 Department of Endodontics, Faculty of Oral and Dental Medicine, Future University in Egypt, Cairo, Egypt; 2 Department of Oral Technology, School of Dentistry, University of Bonn, Bonn, Germany; 3 Department of Oral and Craniofacial Health Sciences, College of Dental Medicine, University of Sharjah, Sharjah, 27272 UAE; 4 Research Institute of Medical and Health Sciences, University of Sharjah, Sharjah, 27272 UAE; 5 Department of Oral and Maxillofacial Surgery, Faculty of Dentistry, Suez Canal University, Ismaillia, 41522 Egypt; 6 Department of Oral Medicine and Radiology, Faculty of Oral and Dental Medicine, Future University in Egypt, Cairo, Egypt; 7 Department of Orthodontics, Faculty of Oral and Dental Medicine, Future University in Egypt, Cairo, Egypt

**Keywords:** Thermography, Acute pulpitis with apical periodontitis, Acute periapical abscess, Chronic periapical abscess, Periapical inflammatory intraoral lesions

## Abstract

**Background:**

Thermography is a contemporary imaging modality based on acquiring and analyzing thermal data using non-contact devices. The aim of the present study was to assess the validity of thermography, compared with that of the reference-standard, for the diagnosis of periapical inflammatory lesions and to evaluate the temperature ranges for acute pulpitis with apical periodontitis (AAP), acute periapical abscess (AA) and chronic periapical abscess (CA).

**Methods:**

AAP, AA and CA were diagnosed based on clinical and radiographic criteria. Thermographic data were acquired using the FLIR E-5 Infrared Camera. Extraoral thermal images were taken from the front and right and left sides of patients whose mouths were closed, and one intraoral thermal image was taken from the palatal perspective. Agreement in the diagnoses based on the combination of clinical and radiographic assessments and the thermographic evaluation was calculated. The temperature ranges of the three diagnostic subgroups were also measured.

**Results:**

A total of 80 patients were enrolled in this study. The mean intraoral thermal image temperature for AA was 37.26 ± 0.36, that for CA was 35.03 ± 0.63 and that for AAP was 36.07 ± 0.45. The differences between the mean intraoral thermal temperatures of the three diagnostic groups were statistically significant (*P* < 0.001). The result of the Kappa coefficient of agreement between the combination of clinical and radiographic assessments and the thermographic evaluation was significant (*P* < 0.001).

**Conclusions:**

Thermography is an effective, quantitative and nonionizing approach that can be used for the diagnosis of periapical inflammatory lesions. The results of the present study indicated that the highest thermal image temperatures were recorded for AA. Thermography might be able to detect inflammatory reactions during the preclinical stage, leading to early diagnosis.

## Background

Periapical inflammatory lesions result from pulpal lesions or disrupted periodontal attachments that extend to the apex, furcation or lateral canals. Periapical lesions are usually consequences of the vascular and anatomic connections between the pulp and periodontium. A thorough history and careful clinical and radiographic examinations are required to diagnose such lesions and develop a treatment plan. However, the diagnosis of periapical inflammatory lesions is always a challenge, even for experienced dental practitioners, due to the multifactorial origin of these lesions, which include diverse interactions between different strains and species of microbes [1].

Thermography is a contemporary imaging modality with various potential applications in dentistry. The technology is based on the acquisition and analysis of thermal data using noncontact devices, and it depends on infrared electromagnetic radiation, which is emitted by objects with temperatures above absolute zero [2]. Temperature differences can be charted on a two-dimensional image, and accordingly, the temperatures of individual spots in an area of interest at a certain time can be recorded [3].

Human tissues are ideal emitters of infrared radiation at room temperature. It has been reported that most of the infrared radiation emitted by the human body has long wavelengths (8–15 µm). Infrared cameras can convert the emitted radiation into electrical signals, which can then be displayed using colors to represent temperature values. Ultimately, a quantitative temperature map of the area of interest is produced, and this map can be utilized to identify different pathological conditons [4].

Thermography has been used in different applications in dentistry. Chronic orofacial pain and temporomandibular disorders were successfully classified thermally [5, 6]. Thermography has been used to evaluate thermal insults to the dental pulp. Thermal image analysis showed that the pulpal temperature increased significantly following the electrothermal debonding of orthodontic brackets, confirming the high risk to the pulp [7]. Thermophotonic lock-in imaging using photothermal wave principles has been used to detect early dental carious lesions [8]. Moreover, thermography has been used successfully to detect malignancies of human hard and soft tissues [9, 10].

The aims of the present study were to assess the validity of thermography for the diagnosis of periapical inflammatory lesions and to compare it with the reference-standard, X-ray radiographs. The temperature ranges for acute pulpitis with apical periodontitis lesions and acute and chronic periapical abscess lesions were evaluated.

## Methods

The study was conducted on patients who were treated at the Oral Diagnosis Clinic at the Faculty of Oral and Dental Medicine, Future University, Egypt, between September 2017 and March 2020. All the patients underwent comprehensive dental, intraoral, and extraoral diagnostic examination procedures. Patients were included in the study if they had any signs or symptoms of swelling, tenderness with palpation, pain with percussion, widening of the lamina dura or apical radiolucency related to any maxillary tooth except the third molars. The exclusion criteria included an age under 18 years or over 60 years; inflammatory conditions of the oral mucosa; severe cognitive, mental and locomotive deficiencies; physiological or pathological conditions associated with changes in thermal homeostasis; smoking habits and the use of nonsteroidal anti-inflammatory drugs. All the patients were informed of the aim of the study, and they signed a formal consent form before participation. Ethical approval for this study was obtained from the Research Ethics Committee at Future University, Egypt. The guidelines of the Declaration of Helsinki were followed in this investigation.

AAP, AA and CA were diagnosed based on clinical and radiographic criteria (Table 1). The inflammatory lesions were related to maxillary teeth except the third molars. The clinical examination involved the observation of decayed and broken restorations, fracture lines, swelling and sinus tracks. Tenderness of the apical area upon palpation, percussion tests, pulp sensitivity tests and tooth mobility evaluations were also performed. The radiographic diagnosis was based on the identification of areas of radiolucency on periapical radiographs. The clinical and radiographic examinations were performed by two calibrated investigators with over 10 years of clinical experience.

**Table 1:** The clinical and radiographic criteria used to diagnose AA, CA and AAP

	AA	CA	AAP
Clinical criteria	Throbbing and pain to light pressure, biting, touching and percussion accompanied by tenderness to palpation and mobility of the tooth. Intra-oral and/or extra-oral swelling may be present	Presence of a draining sinus on the oral mucosa or occasionally on the facial skin not usually associated with pain	Spontaneous pain, extreme sensitivity to heat and cold, awakening at night and considerable tenderness to percussion and pressure on the tooth
Radiographic criteria	No evidence of periapical changes or a slight thickening of the periodontal ligament space	Periapical radiolucent area related to the apex of the tooth	Widening of the periodontal ligament space and loss of lamina dura around the apex

### Thermographic investigation

Thermographic data were acquired using the FLIR E-5 Infrared Camera with MSX (FLIR Systems, Oregon, USA). Images were stored on a memory card in the TIFF file format and processed using specialized software (FLIR Tools Thermal Analysis and Reporting; Desktop, FLIR Systems, Oregon, USA). The camera was calibrated considering the emissivity parameters of the human body (ε = 0.98 and λ˃2 μm) and the ambient conditions (humidity and temperature), and a thermal range from 20 to 250 °C ± 2 °C, a pixel infrared resolution of 10,800 (120 × 90), and adequate shade were selected for optimal color registration. A standard protocol was used by positioning all the electronic devices at least 2 m from the patient, covering windows and reflective surfaces with opaque dark textiles and turning off artificial light sources. Room temperature was set to 20 ± 1 °C, and the air currents were directed away from the patient and toward the periphery of the room. The patients were asked to refrain from smoking, eating and drinking for 60 min prior to the examination, and they remained at rest for 10 min before the procedure. The oral cavity was cooled by rinsing with cold water (10 °C) for 1 min.

**Fig. 1:**
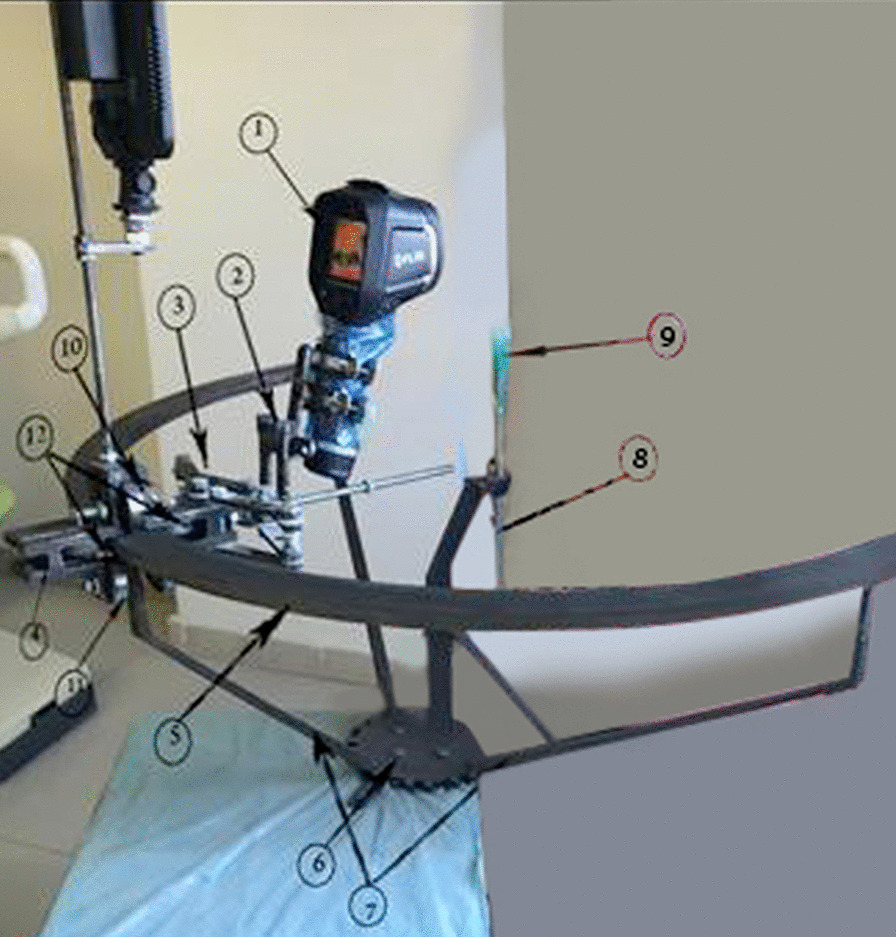
Profile-guided thermography scanner showing: 1. Infrared camera, 2. Adjusting angles, 3. Scissors mechanism, 4. Bearing housing, 5. Guiding profile, 6. Fixing flange, 7. Guide supports, 8. Chin rest support, 9. Chin rest, 10. Upper bearing, 11. Lower bearings, 12. Side bearings

A profile-guided thermography scanner was designed to provide a guided trajectory for the infrared camera using a machined profile, which was designed as an offset contour of the human mandible to maintain the camera in a position such that it faced the patient. Bearing housing was designed to roll on the guide holding the camera, and upper and lower bearings were used to hold the positioning mechanism and the camera, while side bearings were used to adjust the camera angle to face the patient (Fig. 1).

**Fig. 2:**
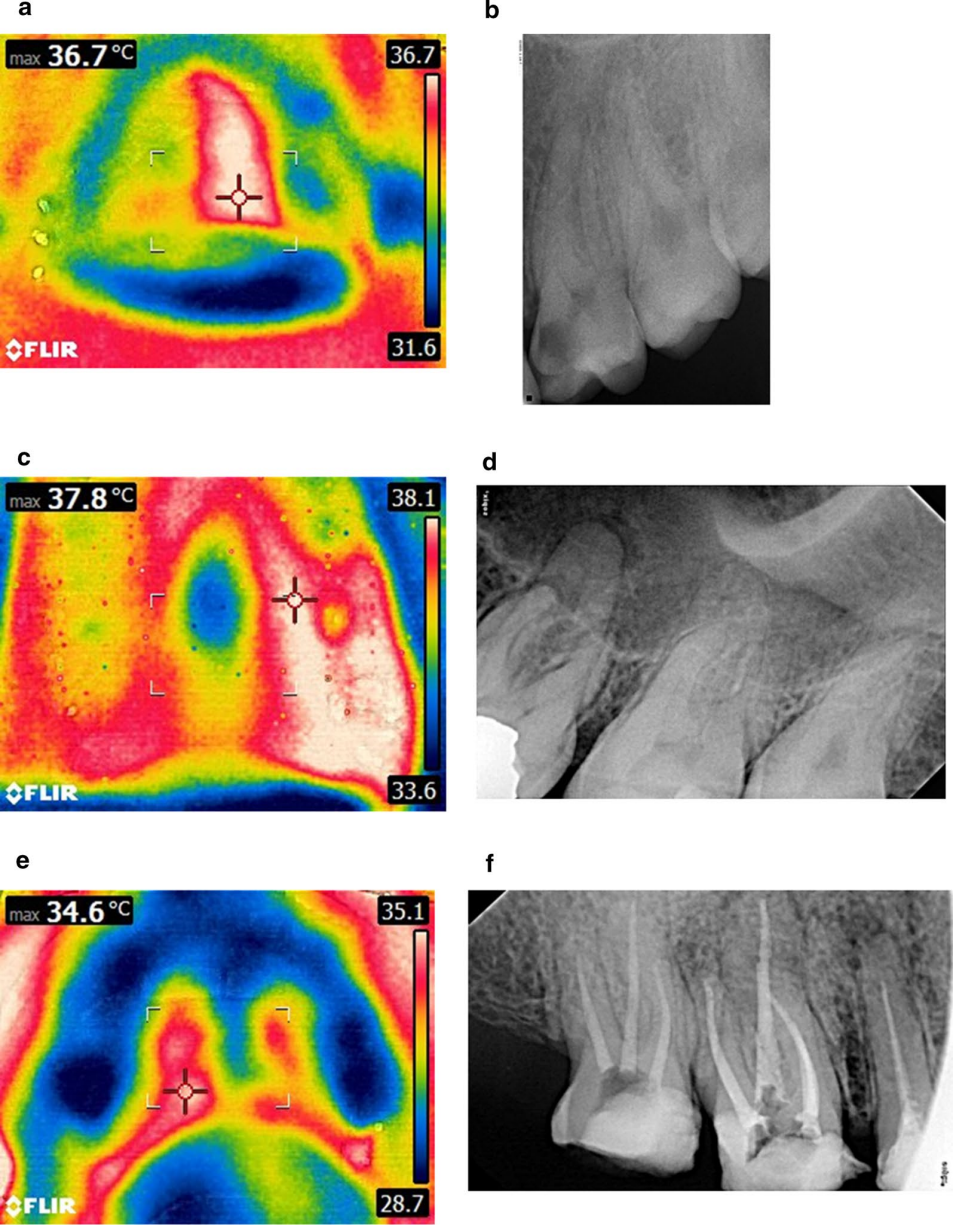
**a** Thermal image for AAP related to the upper left first molar. The heat detector is pointing to the highest temperature (36.7 °C). **b** Periapical radiograph showing widening of the lamina dura around the mesial root of the same tooth. **c** Thermal image for AA related to the upper left first molar with the highest temperature (37.8 °C) detected using Flir Tools software. **d** Periapical radiograph showing widening of the lamina dura around the roots of the same tooth with radiolucency related to the apex of the roots. **e** Thermal image for CA related to the upper right second premolar. **f** Periapical radiograph showing radiolucency surrounding the apex of the upper right second premolar. A sinus tract related to the same tooth was detected on clinical examination

Patients rinsed their mouths with cold water then three extraoral thermal view images were taken from the front and right and left sides of patients whose mouths were closed. The procedure was repeated for five times at one-minute intervals. One intraoral thermal image was then taken from the palatal perspective after 5 min at a distance of 20 cm (Fig. 2).

Thermographic data recording was performed by a third investigator who was blinded to the clinical and radiographic patient records. Agreement in the diagnoses of AAP, AA and CA based on the combination of clinical and radiographic assessments and the thermographic evaluation was calculated to assess the validity of thermography. The temperature ranges of the three diagnostic subgroups were also measured.

### Statistical analysis

The data analysis was performed using the Statistical Package for Social Sciences (SPSS) version 16.0 (SPSS Inc., Chicago, IL, USA). The thermography results are expressed as the mean ± standard deviation (SD). Analysis of variance (ANOVA) was used for testing the null hypothesis that the mean of the groups are equal, followed by the Bonferroni method for multiple comparisons. The Kappa coefficient of agreement was used to assess the relation between the combination of clinical and radiographic assessments and the thermographic evaluation following the conversion of the quantitative data to dichotomous categorical data. P-values of < 0.05 were considered statistically significant.

## Results

A total of 80 patients (42 females and 38 males) were enrolled in this study. Twenty-seven patients with a mean age of 37.37 ± 9.97 years were diagnosed with AA, 29 patients with a mean age of 39.1 ± 12.34 years were diagnosed with CA, and 24 patients with a mean age of 36.96 ± 10.98 years were diagnosed with AAP.

**Fig. 3:**
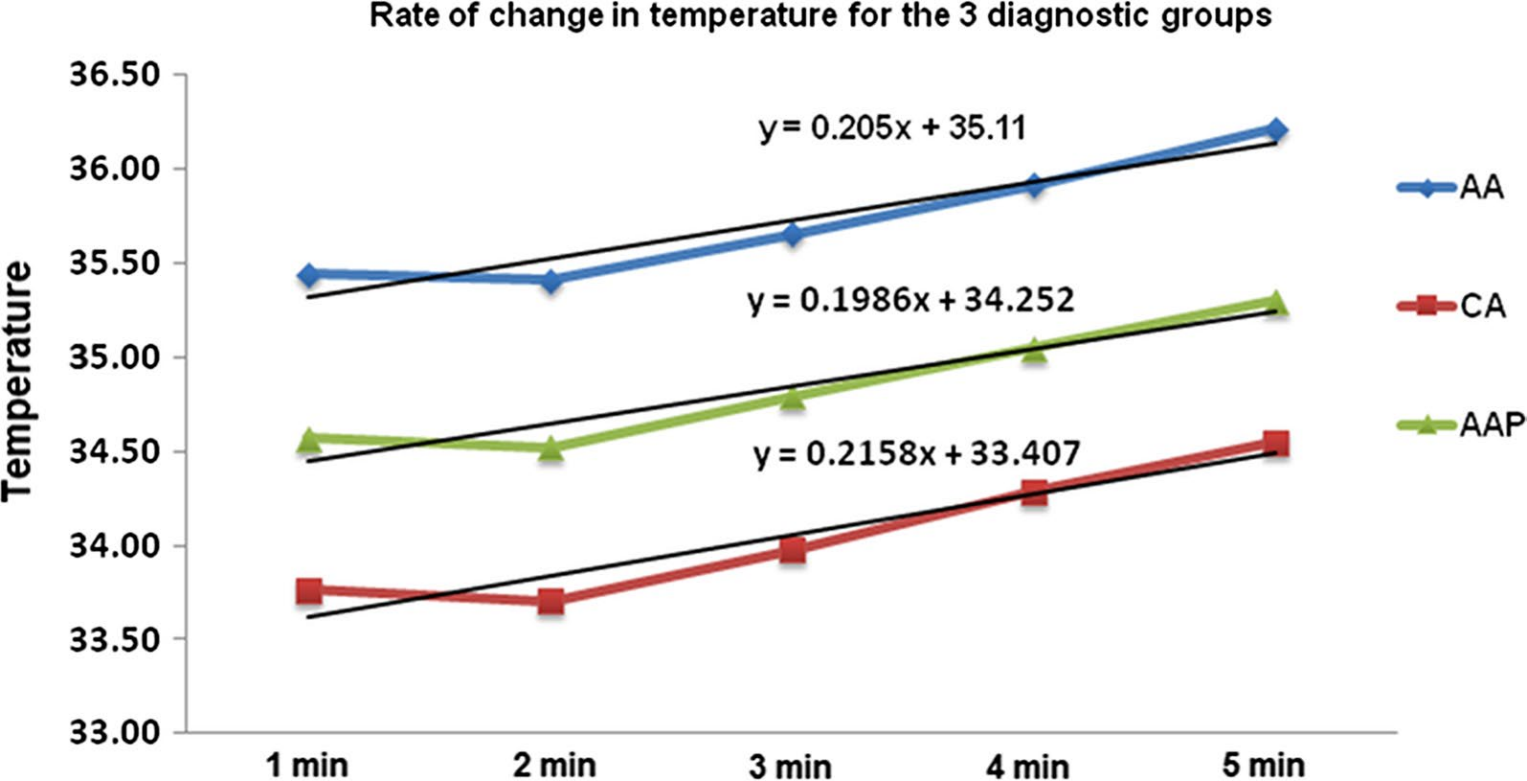
Rates of change in extraoral thermal temperature for the 3 diagnostic groups represented as the intercept of the regression line equation

The mean intraoral thermal image temperature for AA was 37.26 ± 0.36, that for CA was 35.03 ± 0.63 and that for AAP was 36.07 ± 0.45. The rates of change in extraoral thermal temperatures with time for the 3 diagnostic groups are shown in Table 2 and Fig. 3. The results of the analysis of variance (ANOVA) for the assessment of between-group differences were statistically significant (*P* < 0.001). The differences between the mean thermal image temperatures of the three diagnostic groups were statistically significant (*P* < 0.001) (Table 3).

**Table 2:** Comparison of the rate of change in extraoral thermal temperatures with time for the three diagnostic groups

	N	Mean	SD	SEM	95% confidence interval for mean		Minimum	Maximum
					Lower bound	Upper bound		
**AA**								
Temp. After 1 min	81	35.44	0.64	0.07	35.30	35.58	33.90	37.02
Temp. After 2 min	81	35.41	0.59	0.07	35.28	35.54	33.70	37.00
Temp. After 3 min	81	35.65	0.61	0.07	35.52	35.79	33.90	37.50
Temp. After 4 min	81	35.91	0.62	0.07	35.77	36.05	34.00	37.60
Temp. After 5 min	81	36.21	0.71	0.08	36.06	36.37	34.10	37.80
**CA**								
Temp. After 1 min	87	33.76	1.32	0.14	33.48	34.04	27.10	35.82
Temp. After 2 min	87	33.70	1.34	0.14	33.42	33.99	27.10	35.70
Temp. After 3 min	87	33.97	1.08	0.12	33.74	34.21	27.80	35.80
Temp. After 4 min	87	34.29	0.78	0.08	34.12	34.45	31.30	35.90
Temp. After 5 min	87	34.55	0.71	0.08	34.40	34.70	32.50	36.10
**AAP**								
Temp. After 1 min	72	34.57	0.69	0.08	34.41	34.73	32.80	35.88
Temp. After 2 min	72	34.52	0.67	0.08	34.37	34.68	32.90	35.90
Temp. After 3 min	72	34.79	0.63	0.07	34.65	34.94	33.50	36.10
Temp. After 4 min	72	35.05	0.61	0.07	34.91	35.19	33.60	36.60
Temp. After 5 min	72	35.30	0.59	0.07	35.16	35.44	33.90	37.10

**Table 3:** Differences between the mean values of the intraoral thermal images’ temperatures of the three diagnostic groups

Investigators’ diagnosis		Mean difference	SE	*P* value	95% confidence interval	
					Lower bound	Upper bound
AA	CA	2.2	0.1	< 0.001	1.9	2.6
AA	AAP	1.2	0.1	< 0.001	0.8	1.5
AAP	CA	1.0	0.1	< 0.001	0.7	1.4

The Kappa coefficient of agreement (0.970) between the combination of clinical and radiographic assessments and the thermographic evaluation was significant (*P* < 0.001).

## Discussion

Thermography is an imaging modality with a promising future in dental medicine. There are many advantages of thermography, including the possibility of evaluating different diseases without the use of ionizing radiation, which prevents many of the harmful effects of radiation and allows disease evaluation during pregnancy. In addition, thermography is a noncontact technology and is optimal for infection control. Thermography allows the comparison of different areas of interest on two-dimensional images and allows the evaluation to be conducted in real time [11]. The concept behind the use of thermography to aid in the diagnosis of pathologies is based on the fact that heat is always generated in response to inflammation, and thermography images are able to quantify the surface temperature, thus providing an assessment of the microcirculation in a specific area. It has been reported that a temperature difference of more than 3 °C usually indicates infection [12]. Furthermore, thermography is able to distinguish between different infections based on the range of temperature increases associated with each [13]. In the present study, the temperature ranges for each diagnostic group were identified, and the differences between the mean temperatures of the three diagnostic groups were statistically significant. In another study that evaluated the thermal image temperatures for facial cellulitis and dental abscess, a significant temperature difference between the two pathologic conditions was observed. The study reported the temperature differences between affected and contra-lateral unaffected side for abscess as 1.49 ± 1.0 and for cellulitis as 2.4 ± 1.9. The higher differences in facial cellulitis compared to dental abscess were attributed to the wider spread and more tissue damage caused by cellulitis. However, the authors reported a small sample size as a limitation of their study [14]. Other studies reported the efficiency of thermography in classifying chronic orofacial pain and in diagnosing temporomandibular disorders. The thermal classification of orofacial pain resulted in 92% agreement with the clinical diagnosis [5]. Internal derangement and osteoarthritis of the temporomandibular joint showed a temperature increase of + 0.4 °C compared to normal joints [6]. Thermography was also useful to confirm the thermal risk associated with the electro-thermal debonding of orthodontic brackets which resulted in an increase of the temperature of the pulp from 16.8 to 45.6 °C [7]. Inferior alveolar nerve deficit was successfully evaluated thermographically as it showed a temperature increase of + 0.5 °C when compared to normal controls [5].

The capability of thermography for distinguishing the temperature ranges of different pathological conditions can be attributed to recent advancements in technology. The new generation of high-resolution cameras has the ability to detect even minor temperature changes resulting from different pathological conditions. These changes can result from the distinctive pathophysiological pathways of the different pathologies, which might still have a general inflammatory origin. At the beginning of the inflammatory process, the somatic-vegetative reflex of the adrenal gland is activated. This is usually followed by the secretion of histamine and kinins, which lead to local decreases in vascular resistance. Liver metabolic processes then produce acute phase proteins and clotting cascade proteins, leading to muscle protein proteolysis and fever. Increased permeability of the blood vessel endothelium, which leads to a movement of water to the perivascular space, usually occurs within the first hour following stimulation [15]. Edema and reddening usually follow due to the difficulty of capillary blood outflow from the site of inflammation, leading to passive hyperemia. The secretion of endogenic pyrogens such as interleukin 1 and tumor necrosis factor, excitation of the sympathetic system and increase in adrenal glucocorticoid levels lead to a febrile state. These changes, together with changes in the metabolic state of the inflammatory site, lead to a local increase in temperature [16].

The gradual increase in thermographic temperatures has been shown to be linked to the gradual reduction in the cooling effect of rinsing with cold water. After 5 min, the thermographic temperatures are expected to reflect the increase in the concentrations of inflammatory mediators, which peak 96 h after the start of inflammation [17]. The results of the present study support the hypothesis that thermography might be able to detect the inflammatory reaction during the preclinical stage, leading to early diagnosis [18]. Thus, digital infrared imaging may be able to promote the early diagnosis of inflammatory conditions, even before clinical symptoms are observed [19].

The reported control temperature for the maxillary oral mucosa was 32.2 °C, which makes it unlikely to have false positive results considering the results of the present study [20]. However, the use of thermography in dentistry still suffers from several limitations including the high price range for quality cameras, the inaccurate results in patients suffering from sunburns and facial scars and the technique sensitivity, which results in reduction in the images resolution if the angle and the distance are not adjusted [20].

## Conclusions

Thermography is an effective, quantitative and nonionizing approach that can be used for the diagnosis of AAP, AA, CA. The results of the present study indicated that the highest thermal image temperatures were recorded for AA. Thermography might be able to detect inflammatory reactions during the preclinical stage, leading to early diagnosis.

## Data Availability

The datasets used and/or analysed during the current study are available from the corresponding author on reasonable request.

## Abbreviations

AAP: Acute pulpitis with apical periodontitis
AA: Acute periapical abscess
CA: Chronic periapical abscess
